# Tides of Blockchain in IoT Cybersecurity

**DOI:** 10.3390/s24103111

**Published:** 2024-05-14

**Authors:** Love Allen Chijioke Ahakonye, Cosmas Ifeanyi Nwakanma, Dong-Seong Kim

**Affiliations:** 1 ICT-Convergence Research Center, Kumoh National Institute of Technology, Gumi 39177, Republic of Korea; loveahakonye@kumoh.ac.kr (L.A.C.A.); cosmas.ifeanyi@kumoh.ac.kr (C.I.N.); 2 IT Convergence Engineering, Kumoh National Institute of Technology, Gumi 39177, Republic of Korea

**Keywords:** BaaS, blockchain, IoT, intrusion detection, smart contracts, security

## Abstract

This paper surveys the implementation of blockchain technology in cybersecurity in Internet of Things (IoT) networks, presenting a comprehensive framework that integrates blockchain technology with intrusion detection systems (IDS) to enhance IDS performance. This paper reviews articles from various domains, including AI, blockchain, IDS, IoT, and Industrial IoT (IIoT), to identify emerging trends and challenges in this field. An analysis of various approaches incorporating AI and blockchain demonstrates the potentiality of integrating AI and blockchain to transform IDS. This paper’s structure establishes the foundation for further investigation and provides a blueprint for the development of IDS that is accessible, scalable, transparent, immutable, and decentralized. A demonstration from case studies integrating AI and blockchain shows the viability of combining the duo to enhance performance. Despite the challenges posed by resource constraints and privacy concerns, it is notable that blockchain is the key to securing IoT networks and that continued innovation in this area is necessary. Further research into lightweight cryptography, efficient consensus mechanisms, and privacy-preserving techniques is needed to realize all of the potential of blockchain-powered cybersecurity in IoT.

## 1. Introduction

The Internet of Things (IoT) has revolutionized computing and sensing across various domains, offering ubiquitous connectivity and data exchange [1,2]. With industrial networks embracing IoT on a massive scale, the interconnectedness of billions of devices presents both opportunities and challenges [3]. This proliferation amplifies the potential for vulnerabilities and intrusions, significantly expanding the attack surface. Consequently, continuous monitoring becomes imperative to detect and mitigate security threats swiftly. As IoT deployments continue to burgeon, ensuring the resilience of networks against cyber attacks demands vigilant surveillance and proactive measures to safeguard sensitive assets and critical infrastructure [4].

The IoT system’s security and privacy could be improved with the help of blockchain technology [2,5], with open, unchangeable, and safe transactions [5], thereby enhancing security and privacy by utilizing blockchain technology [6]. Integrating IoT ecosystems with blockchain technology is a promising strategy to fortify cybersecurity measures in the evolving digital landscape. This introduction elucidates the pivotal role of blockchain in enhancing IoT cybersecurity, emphasizing its transformative capabilities and urgent relevance in contemporary digital environments. As IoT systems proliferate and interconnect, leveraging blockchain presents a compelling solution to address the escalating cybersecurity challenges. By elucidating the symbiotic relationship between IoT and Blockchain, this discourse underscores the imperative for integrating these technologies to safeguard digital assets and mitigate emerging threats effectively.

Artificial intelligence (AI) algorithms are essential for detecting intrusions and attacks in IoT, providing advanced capabilities to secure IoT ecosystems [7]. These algorithms apply machine learning (ML) techniques to analyze real-time data from IoT devices and identify anomalies and prospective security threats. By constantly observing network activity, sensor data, and device behavior patterns, AI algorithms can identify unusual activities indicative of malicious intent [8,9]. One widely used approach is anomaly detection, where AI models learn normal device behavioral patterns and flag deviations as potential threats [3]. Supervised learning algorithms are frequently employed and trained on labeled datasets to distinguish benign from anomalous conduct. Convolutional and recurrent neural networks (RNNs) are two examples of very effective deep learning techniques. CRNNs are skilled at processing sequential data, which makes them helpful in identifying intricate attack patterns, whereas CNNs examine structured data, such as network traffic [9,10]. In addition, reinforcement learning algorithms can adapt and improve over time by continuously interacting with the IoT environment and learning from feedback to enhance intrusion detection capabilities. AI algorithms offer powerful tools for detecting intrusions and attacks in IoT, enabling proactive security measures to mitigate risks and safeguard IoT networks from cyber threats [11].

Blockchain technology has recently been used by researchers in intrusion detection systems (IDSs) for improved monitoring and detection, prevention of malicious activities or attacks, and tamper-proof transactions and storage in IoT devices and networks [2,12]. Blockchain immutability promotes security and efficient data storage for resource-constrained systems. Despite offering decentralization, scalability, transparency, and immutability, this technique has challenges compared to the existing IDS approaches [5,13]. Over the past decade, the focus on IDS for securing IoT/IIoT has intensified, driven by the need for stringent controls, ensuring real-time operation, data integrity, and compatibility with limited telecommunication protocols [1,3,14]. While existing literature offers insights into risks to security and critical surveillance, this paper provides a comprehensive unconventional survey, explicitly exploring the fusion of blockchain and AI for fortified IoT/IIoT IDS approaches.

Blockchain’s potential for IoT security lies in its decentralization, immutability, transparency, and smart contracts. Integrating it with ML, encryption, and identity management systems strengthens IoT security frameworks [15,16]. Industry interest and advancements in blockchain align with current research priorities, but gaps exist in understanding its full potential and scalability [15]. Analyzing blockchain’s current state and future trends guides informed decisions and advancements, making it a crucial aspect of IoT security evolution [15]. This study explores the intersection of blockchain and IDS for IoT cybersecurity, as blockchain’s features position it to enhance IoT security.

This article outlines blockchain research opportunities for IoT network cybersecurity, trends, future direction, and challenges. First, we conduct a background study on IoT and an overview of blockchain technology to provide context and clarity, followed by its unique features and challenges, motivating a general-purpose IDS. We propose an IDS and define its relationship to blockchain technology, forming a comprehensive blockchain-based IDS framework. The presented results demonstrate the role of blockchain in improving intrusion detection performance. Finally, we discuss research opportunities framed by our blockchain-integrated IDS. This article provides a framework within which researchers can better engage and collaborate in studying and exploring next-generation IDS for IoT networks with a vision toward ubiquitous, scalable, transparent, immutable, and decentralized IoT networks. Notably, this review makes the following significant contributions:

1. Utilizing the preferred reporting items for systematic reviews and meta-analyses (PRISMA) article collection approach, this study systematically gathers articles on AI, blockchain, IDS, IoT, and IIoT, shedding light on challenges, trends, and emerging review areas in IoT IDS designs for security.
2. Focusing on articles published between 2019 and 2024, this review captures recent advancements in AI and blockchain-based IDS designs, ensuring relevance and currency of insights.
3. Evaluation of various AI blockchain integration techniques prioritizes factors like fidelity, transparency, immutability, robustness, and compactness, providing a nuanced understanding of their performance.
4. This study underscores blockchain’s pivotal role in fortifying IoT/IIoT security measures by showcasing its efficacy in enhancing intrusion detection performance.

## 2. Background Study

This section provides an essential framework for a clearer understanding of the discussion by elucidating the trends, limitations, and opportunities in IoT. It also provides concepts pertinent to the current investigation of blockchain applications.

### 2.1. Exploring Evolving Opportunities, Trends, and Striking Demands in the Internet of Things

The IoT represents a significant breakthrough in connectivity by connecting billions of internet-enabled devices, fostering intelligent interactions, and integrating physical infrastructure with digital systems. IoT has expanded to encompass various industries, including smart factories, healthcare, smart cities, and transportation [17]. Recent forecasts indicated that IoT-connected devices exceeded 10 billion in 2021, and are expected to reach 41 billion by 2027, with smart home and factory devices driving the market [18]. Sensors and actuators are vital to the Internet of Things as they gather data and manipulate actual environments. However, IoT faces challenges like security vulnerabilities resulting from resource constraints in sensor nodes and interoperability problems caused by diverse communication protocols. Despite these obstacles, IoT’s convergence with data analytics and AI enables real-time decision-making and predictive maintenance, leading to significant process improvements. To ascertain the potentialities of IoT, it is necessary to address privacy, security, data heterogeneity, and interoperability concerns.

**Figure 1:**
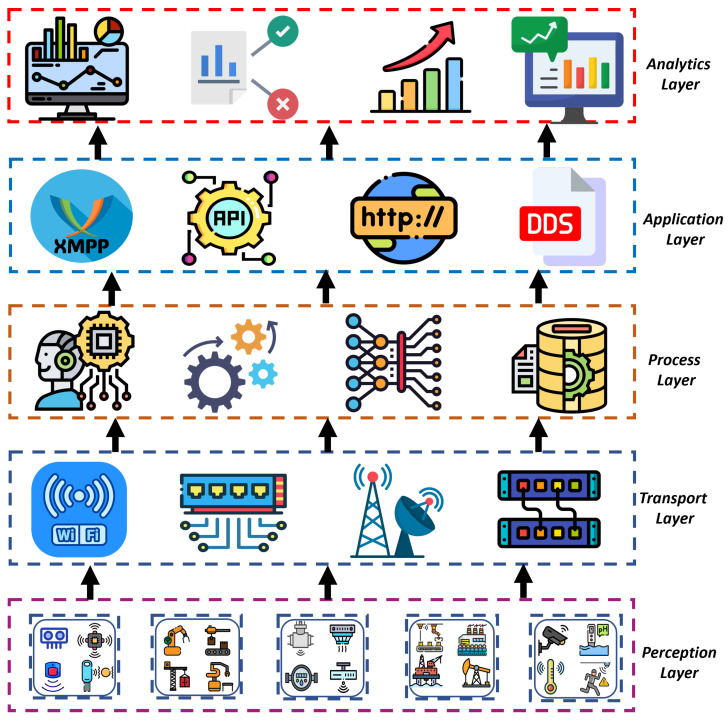
IoT architectural layers.

The proliferation of IoT devices, the diverse nature of these devices, and the evolution of communication protocols have led to a surge in enabling technologies from an engineering standpoint [19]. AI and ML techniques have further enhanced the potential of IoT by extracting insights from heterogeneous sensor data, thereby reshaping business operations [18,20]. The modular design of IoT systems, which abstracts these systems into separate components, enhances their adaptability and clarifies their architecture [20]. As in Figure 1, the layered structure of IoT comprises the perception, transport, processing, application, and analytics layers. The perception layer encompasses physical devices that sense the environment and communicate data to higher layers, while the transport layer facilitates communication between devices and cloud-based services. The processing layer, typically hosted on edge or cloud platforms, provides storage and computation capabilities, enabling scalability and interoperability. The application layer governs system operations, interacts with users, and manages logical processes. Finally, the analytical layer offers users actionable insights, enhancing decision-making. However, security challenges persist, particularly in resource-constrained IoT devices and cloud-based middleware, highlighting the need for robust security measures [20].

The IoT conceptual framework links billions of devices with internet access, allowing data to interact with each other and their environment. The evolution of IoT has led to ubiquitous data access, enabling real-time connectivity and interaction between physical and digital systems across various domains. From its origins supporting radio frequency identification (RFID) technology, IoT has expanded into diverse healthcare, transportation, and smart factory/city applications [21]. Recent statistics indicate a significant rise in connected IoT devices, with projections reaching 41 billion by 2027, translating to over 152,000 new connections per minute by 2025 [22,23]. This growth reflects a booming market, with the global IoT market reaching USD 157.9 billion in 2021, primarily driven by industrial applications and intelligent devices [22,23].

IoT presents opportunities to enhance productivity through real-time asset monitoring and control. Industries make informed decisions by leveraging data from IoT devices, such as sensors and actuators, improving operational efficiency. Additionally, IoT facilitates the development of smart applications in various sectors like factories, homes, cities, and agriculture, leading to increased convenience and efficiency in daily operations.

### 2.2. IoT/IIoT Vulnerabilities and Attacks

Security remains a paramount concern in IoT and IIoT, given the potential safety and risks associated with compromised systems. Vulnerabilities persist due to protocol limitations, insufficient mitigation strategies, and challenges in real-time monitoring, as highlighted by various studies [24,25,26,27]. Attacks target various substrata of the IoT framework, often aligning with the Open Systems Interconnection (OSI) model [25,26].

A comprehensive examination of SCADA attacks reveals their significance within the IIoT [24]. SCADA is a pivotal component of the IIoT infrastructure responsible for monitoring industrial processes. SCADA systems integrate data acquisition, transmission, and human–machine interface (HMI) techniques. HMIs serve as interfaces connecting individuals to devices, facilitating data visualization and real-time monitoring of production processes, as well as machine input and output. SCADA architecture typically comprises the master terminal unit/station (MTU/MSU), which functions as the control center, sub-MSUs/sub-MTUs as secondary control centers, remote terminal units/remote station units (RTUs/RSUs) acting as programmable logic controllers (PLCs) and intelligent end devices (IEDs), used for sensor and actuator data monitoring. The authors looked at common IoT/IIoT attacks, their methodologies, and frequently employed tools in IoT/IIoT [24,28] as follows:

1. Distributed denial-of-service and denial-of-service (DDoS/DoS) attacks deprive authorized users of network resources, primarily targeting availability requirements [4]. In such scenarios, a compromised remote terminal unit (RTU) inundates the master terminal unit (MTU) with arbitrary packets, causing network capacity depletion and hindering the accessibility of resources for legitimate users. The RTU and MTU’s communication ability is interfered with, impeding supervision and process tracking. Low Orbit Ion Cannon (LOIC), Slowloris, Raksmart, Hulk, and Tor’s Hammer are typically used attack tools [24,29,30].
2. The man-in-the-middle (MiTM) attack intercepts network traffic by infiltrating device communication paths. It is achieved by observing the network, inserting irregularities into transmissions, and relaying them to the intended recipient. Successful execution of this attack hinges on maintaining the session connection while keeping the attacker’s presence concealed, using spoofed IPs to evade detection [28,31]. SSLStrip, Evilgrade, and Ettercap are standard tools that enable MiTM attacks [24,32,33].
3. MTUs and sub-MTUs can access the wired or wireless network through passive or active eavesdropping, allowing attackers to introduce spyware and exploit vulnerabilities [28,34].
4. Masquerade attacks involve impersonating legitimate network users through fake identities and IP spoofing, facilitating the theft of sensitive information. Attackers may employ brute force tactics to exploit stolen passwords for unauthorized access [28,35].
5. Viruses, Trojan horses, and worms are deployed by attackers post-MitM or masquerade attacks to infect MTUs. These malicious codes grant unauthorized access to the infected system, allowing attackers to launch further assaults or propagate throughout the network, potentially causing system instability or collapse [28].
6. Fragmentation attacks exploit weaknesses in packet reassembly processes, causing MSU/MTU failure when transmitting oversized data, leading to system collapse [28].
7. Doorknob rattling involves preparatory actions, such as limited system access attempts, to test security measures’ readiness and responsiveness before an attack [28].
8. Attacks known as reconnaissance aim to learn more about a network and its hardware characteristics. Guarding sensor readings from the operational procedure is, therefore, essential. Attacks such as response injection introduce deceptive inputs into a control system, prompting control algorithms to make incorrect decisions. In a command injection assault, fictitious control commands infiltrated the control system. Human intervention may cause an improper control action, or bogus commands may be injected and cause RTU and field device register values to be overwritten [24,28,36].

### 2.3. Overview of Blockchain Technology

A blockchain, originally designed to support cryptocurrencies, has evolved into a disruptive force across various sectors. It serves as a decentralized ledger, ensuring secure and transparent recording of transactions. Its distributed architecture and cryptographic principles guarantee data integrity and resilience against tampering [37]. Security issues with IoT devices, blockchain, and connecting IoT devices to blockchain networks are cybersecurity concerns for blockchain-based IoT systems [2,38].

**Figure 2:**
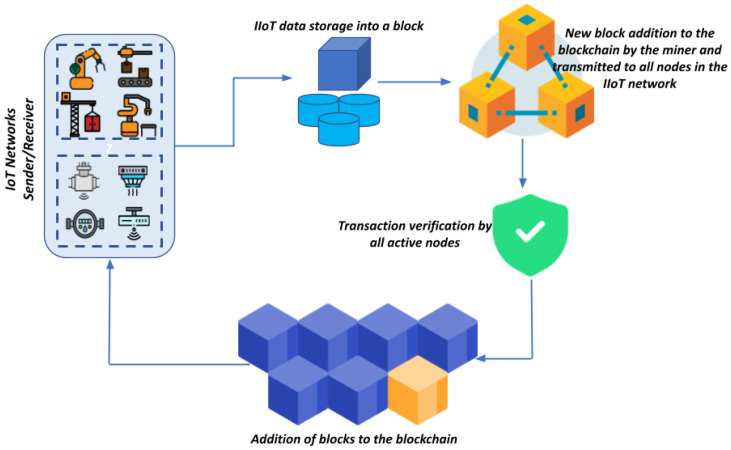
Blockchain transaction process.

A blockchain operates as an open digital ledger on a peer-to-peer network, recording timestamped transactions in immutable blocks. Each block is connected to the following and encrypted, maintaining transparency and integrity without centralized control. It encompasses public and private variants, with public blockchains offering universal access and private ones restricting entry to authorized entities [38]. Transactions undergo digital signing, grouping, and storage in a distributed electronic database, ensuring consensus and verification to prevent tampering. This decentralized approach ensures data consistency across all ledger copies. Figure 2 shows the blockchain data transmission approach within the IoT networks.

#### 2.3.1. Major Blockchain Security Features

1. Data immutability and integrity: Blockchain’s immutability guarantees that recorded data remain unchangeable without network consensus, making it ideal for securing critical IoT data like sensor readings, supply chain details, and device logs. This feature is crucial for maintaining data integrity, a top priority in IoT systems requiring accurate and unaltered data throughout storage and transmission [2,39].
2. Decentralization and transparency: Acting as decentralized and distributed ledgers, transactions are recorded across numerous nodes, ensuring no single entity controls the network. The decentralized architecture in IoT devices lessens the dependence on central authorities and promotes transparent and tamper-resistant transactions. It eliminates single points of failure and bolsters system resilience against cyber threats [2,40].
3. Smart contracts: These self-executing agreements coded on the blockchain automatically execute actions based on conditions, reducing reliance on intermediaries in IoT transactions [41,42]. By automating predefined tasks, such as maintenance alerts or data validation, smart contracts improve efficiency and minimize the need for intermediaries and potential vulnerabilities in IoT transactions [40].
4. Consensus mechanisms: Consensus mechanisms are sets of rules and protocols used in blockchain networks to achieve agreement among network participants regarding the validity of transactions and the state of the distributed ledger [43]. This ensures that all nodes in the network reach a consensus or joint decision about the current state of the blockchain. Various consensus mechanisms facilitate agreement and trust in decentralized networks by establishing rules for adding new transactions to the blockchain and resolving conflicts among participants [44]. Some of these mechanisms are as follows:
  a. Proof of Work (PoW): Requires solving complex puzzles for transaction validation and block creation; ideal for highly secure IoT systems like industrial control systems [42,43,44].
  b. Proof of stake (PoS): Selects validators based on staked coins; offers energy efficiency suitable for resource-constrained IoT devices like smart home systems [42,43].
  c. Delegated proof of stake (DPoS): Uses elected nodes for transaction validation, ensuring high speed and scalability for real-time IoT applications like smart cities.
  d. Proof of authority (PoA): Validators verify identity; benefits enterprise IoT deployments like supply chain management and ensures accountability [42,43].
  e. Practical Byzantine fault tolerance (PBFT): Focuses on low latency and high throughput, making it suitable for financial IoT systems or autonomous vehicles requiring rapid consensus. These mechanisms collectively ensure data integrity, security, and trust in IoT, tailored to specific IoT application needs and constraints [42,43,44].
5. Identity management and authentication: Blockchain-based identity solutions enable secure and verifiable identity management in IoT to establish trust among themselves, ensuring that only authorized devices participate in the network [45].
6. Encryption: Transactions and data stored on the blockchain are encrypted using advanced cryptographic algorithms, ensuring that data remain private and secure, and protecting sensitive IoT data against vulnerability [45].
7. Privacy and confidentiality: Private blockchains provide controlled access to data, guaranteeing confidentiality and making them suitable for scenarios where sensitive information needs secure sharing. IoT leverages private blockchains for securely exchanging critical data, such as patient health records or industrial process data [2,45].

**Figure 3:**
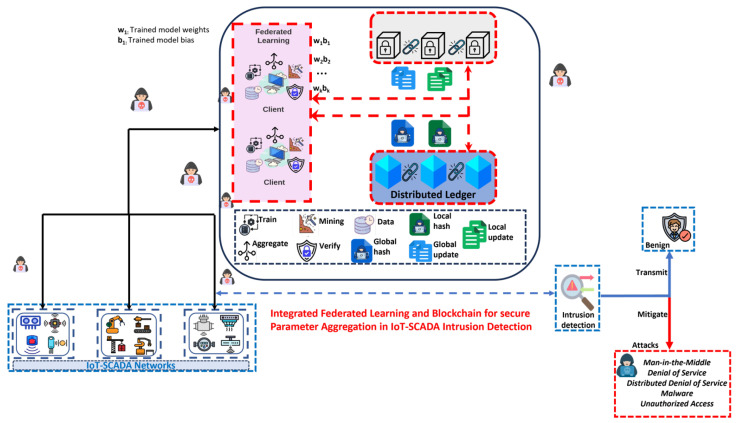
A diagram of an example of the combination of blockchain and IDS, visually demonstrating the benefits of intrusion detection [46].

Blockchain’s features contribute significantly to IoT cybersecurity by providing trust, transparency, and robustness [2,5]. However, scalability remains a hurdle for expansive IoT implementations due to energy-intensive consensus mechanisms. In addition, interoperability is needed across various blockchain and IoT protocols for seamless device integration [5,38]. Researchers continue to explore innovative solutions to address challenges and enhance the synergy between blockchain and IoT. Figure 3 elucidates blockchain integration with AI for intrusion detection.

#### 2.3.2. Opportunities and Challenges in Blockchain–IoT Convergence

The convergence of blockchain and IoT presents a compelling synergy, combining blockchain’s decentralized, transparent ledger with the interconnected network of IoT devices [47,48]. Blockchain, known for its secure transaction data storage, has expanded beyond cryptocurrencies like Bitcoin to include immutable data chains [49]. IoT, conversely, encompasses a vast array of interconnected devices exchanging data, facilitating innovative interactions, and bridging physical and digital domains [47]. Integrating IoT with blockchain networks presents opportunities to enhance data integrity, trust, and decentralization in IoT systems [2,50]. Through blockchain, IoT devices can securely transmit data, ensuring immutable records of transactions [51]. This approach fosters trust between devices, reducing dependence on central authorities and intermediaries, thus enhancing security and resilience [51,52]. The intersection of IoT and blockchain faces challenges in scalability, resource constraints, and privacy [2,21,50,52,53,54,55,56,57]. As IoT expands, ensuring blockchain scalability while considering the limitations of IoT devices becomes crucial [56]. Optimizing blockchain solutions for IoT devices’ computational and storage constraints poses a challenge [58,59]. Balancing transparency and data privacy is also complex, requiring innovative approaches to maintain the benefits of a transparent ledger while safeguarding sensitive information.

#### 2.3.3. Trends and Innovations in Blockchain and IoT Convergence

Several significant trends mark the convergence of blockchain and IoT [48]. Edge computing is rising, boosting real-time processing for IoT devices and reducing latency, while AI integration enables advanced decision-making and predictive analytics [54]. The deployment of high-speed 5G networks further empowers IoT applications [60], complemented by developing communication frameworks like blockchain-enabled architectures to ensure secure data exchange among IoT nodes [21]. However, navigating this landscape requires addressing challenges and seizing opportunities to shape the future of interconnected intelligent systems [21,54].

Blockchain technology has garnered attention for its secure transaction methods and potential to address internet security issues. Recent innovations like federated blockchain offer enhanced scalability and transaction privacy, exemplified by platforms like R3 and Corda [21,61,62]. Blockchain-as-a-service (BaaS) simplifies blockchain app development and maintenance, while Ricardian contracts provide legally valid electronic documents linked to agreements [21,61,63]. Blockchain interoperability facilitates seamless data exchange and cross-chain transactions, while integration into social networking incentivizes content creation through token rewards. Hybrid blockchains offer enhanced security and flexibility by combining public and private blockchain elements [21,61,62,63].

#### 2.3.4. Potential Use and Applications

The IoT industry is witnessing a surge in interest, focusing on integrating real-time data analytics directly into IoT standards. Previously seen as a passive data monitoring tool, IoT now empowers autonomous applications with real-time decision-making capabilities, becoming a fundamental requirement in deployments [5,64]. For instance, integrating real-time analytics with equipment monitoring systems in manufacturing facilities has transformed predictive maintenance practices [2,21]. By combining sensor data with advanced analytics, manufacturers can predict equipment failures proactively, minimizing downtime and optimizing production efficiency, especially in critical industries like automotive manufacturing or semiconductor fabrication [2,64].

Meanwhile, the modern internet landscape is evolving to prioritize the availability and security of connected resources. With its decentralized ledger system, blockchain technology is reshaping transactions across sectors like finance, healthcare, and supply chain management [2,21,51,65,66]. Blockchain enables smart devices to offer users greater control and insights, while integrated sensors enhance real-time monitoring in supply chains [65]. Smart contracts streamline agreement execution, reducing reliance on intermediaries [2]. Furthermore, blockchain applications extend to healthcare, electronic voting systems, digital identity verification, and property registration, addressing governance and asset management challenges [67]. In addressing global challenges such as the COVID-19 pandemic, blockchain, and IoT integration offer transparent and efficient solutions for collective action [2].

The blockchain’s decentralized and impenetrable structure offers several benefits when integrated into IoT systems. Some critical use cases and applications are as follows:

1. Data integrity and immutability: Ensures data integrity from IoT devices, offering an immutable ledger where data records are cryptographically linked, time-stamped, and unalterable. Each transaction is securely stored, guaranteeing the authenticity and reliability of IoT data [2,56,68].
2. Secure device identity and authentication: Verifies IoT device identities and prevents unauthorized access. Blockchain-based digital certificates uniquely identify devices, with smart contracts enforcing access control. Only authorized devices, validated through cryptographic measures, can interact within the network [21,56,66].
3. Decentralized access control: Reduces reliance on central authorities and eliminates single points of failure. Blockchain enables decentralized access control through smart contracts, ensuring distributed permissions management. No single entity controls the entire IoT network, enhancing resilience and security [2,21,56].
4. Supply chain transparency and traceability: Tracks the journey and origin of data and records all transactions transparently and tamper-proof, providing an immutable audit trail. This fosters trust and reduces the risk of anomaly [2,52,56].
5. Secure firmware updates: Ensures secure over-the-air (OTA) updates for IoT devices and verifies the authenticity of firmware updates, allowing devices to validate software integrity before installation. This safeguards against malicious updates and ensures device security [2,21,56].
6. Distributed threat intelligence sharing: Collaborates on threat intelligence across IoT networks and facilitates secure sharing of threat data among devices and organizations. Malware signatures, attack patterns, and other threat intelligence can be exchanged, enhancing collective defense mechanisms [2,21,56].
7. Privacy-preserving data sharing: Enables selective data sharing while protecting privacy, employing privacy-preserving techniques like ZKPs to enable selective data disclosure. Users can share specific data without revealing sensitive information, ensuring privacy while promoting collaboration [2,21,56].
8. Smart contracts for automated security policies: Automates security policies and responses. Smart contracts execute predefined security rules autonomously. For instance, compromised devices can be automatically isolated from the network, preventing further threats and maintaining network integrity [2,51,56].

### 2.4. Examining Blockchain’s Progression in the Quantum Age

Notwithstanding the industrial transformation by blockchain’s decentralized and secure characteristics, the rise of quantum computing presents significant challenges to the conventional cryptographic methods underpinning blockchain systems. Quantum computers exploit quantum bits (qubits) to perform computations exponentially faster than classical computers, potentially breaking widely used encryption algorithms like Rivest–Shamir–Adleman (RSA) and elliptic curve cryptography (ECC) [69,70]. Shor’s algorithm exemplifies this threat by efficiently factoring large numbers and exposing current encryption schemes [71]. Researchers are actively developing post-quantum cryptographic (PQC) algorithms that resist attacks from classical and quantum computers to counter quantum threats [71,72,73]. These PQC algorithms ensure long-term security for blockchain systems, including lattice-based cryptography, code-based cryptography, hash-based signatures, and multivariate polynomial cryptography [69,70].

Blockchain platforms are exploring the integration of post-quantum cryptography to address these challenges [70]. This integration entails upgrading cryptographic primitives such as digital signatures, key exchange protocols, and hash functions to PQC standards while maintaining backward compatibility and transaction efficiency [72]. Adopting proactive measures and fostering collaboration among blockchain developers, cryptographers, and quantum computing experts are crucial for securely navigating this transition [70,71,73]. Implementing robust post-quantum cryptographic standards is paramount to upholding the integrity and security of blockchain systems amidst quantum advancements [69,70,72].

### 2.5. Analysis of Survey on Blockchain for Security Concerns of IoT/IIoT

Ferrag et al. [55] conducted a summary of existing studies on IoT network security with blockchain and investigated blockchain-based security and privacy systems across various types of IoT applications. The work compared consensus algorithms based on nine properties and classified security analysis techniques into four categories. Finally, they highlighted steps for constructing and assessing security systems built on blockchain. Exploring blockchain’s potential beyond cryptocurrency, Padma et al. [53] focused on its applications in IoT monitoring and supply chain management, proposing blockchain integration as a viable solution and highlighting research challenges and opportunities in leveraging blockchain for IoT advancement. Abubakar et al. [57] conducted a thorough survey on integrating blockchain with IoT, outlining limitations, benefits, and architectural insights across various IoT domains, addressing challenges, exploring solutions, and proposing future research directions.

Ankit et al. [38] systematically surveyed recent IoT security technologies, emphasizing session keys, blockchain integration, AI-based authentication, and authentication, stressing the need for ongoing improvements and collaboration in security mechanisms to address evolving threats and inspire future research in IoT security. Blockchain’s decentralized nature offers promising solutions for enhancing IoT security, as explored by Banda et al. [74], who reviewed security enhancements facilitated by blockchain for IoT, including identity management, authentication, and data privacy, and examined its role in enabling end-to-end food traceability. Alam et al. [21] investigated blockchain integration with IoT architecture, evaluated academic research, discussed issues of interoperability and stability, and explored trends and advantages of combining blockchain with IoT.

Mathew et al. [12] analyzed blockchain’s capabilities in IIoT, identifying vulnerabilities and proposing blockchain integration with collaborative IDS to enhance trust in IIoT networks. Shamar et al. [58] concluded that integrating blockchain with IoT presents significant security challenges and emphasized the need for pre-validation of data, security measures in public spaces, and tailored lightweight blockchain-powered solutions for IoT requirements. Alzoubi et al. [56] evaluated the state of blockchain-integrated IoT, examining challenges, proposed solutions, future research directions, and emerging trends, aiding practitioners and researchers in navigating integration complexities. A new paradigm investigated the possibility of handling security and privacy concerns, particularly in IDS for IIoT networks employing a combination of federated learning (FL) and blockchain [59]. Exploring the potential of blockchain in FL for enhancing IDS in monitoring IIoT network traffic offered recommendations for effective implementation. It also discussed concerns and potential avenues for future study in the duo for cybersecurity and intrusion detection for IIoT.

### 2.6. An Overview of Related Works and Areas for Research

The review mentioned above in blockchain and IoT integration shows that the applicability of blockchain to IoT cybersecurity has not yet been thoroughly studied. This evaluation effort boldly depicts the connection between explainable AI (XAI) and IoT security. Although there are surveys on the integration of blockchain and IoT for cybersecurity, as far as we know, research has yet to consider demonstrating the role of blockchain in improving intrusion detection performance. However, despite the multiple surveys regarding the security of IoT and blockchain, a limited survey is available on integrating both technologies for intrusion detection in IoT. Thus, this is the first attempt to comprehensively review incorporating blockchain and IoT for secure intrusion detection in IoT networks. Table 1 summarizes the research on blockchain technology’s role in IoT cybersecurity, focusing on its limitations and the advancements proposed in this research. Existing studies offer valuable insights into how blockchain can enhance IoT security, particularly in IDS. This research guides future investigations, suggesting avenues for further integration and exploration in this field.

## 3. Review Methodology

**Figure 4:**
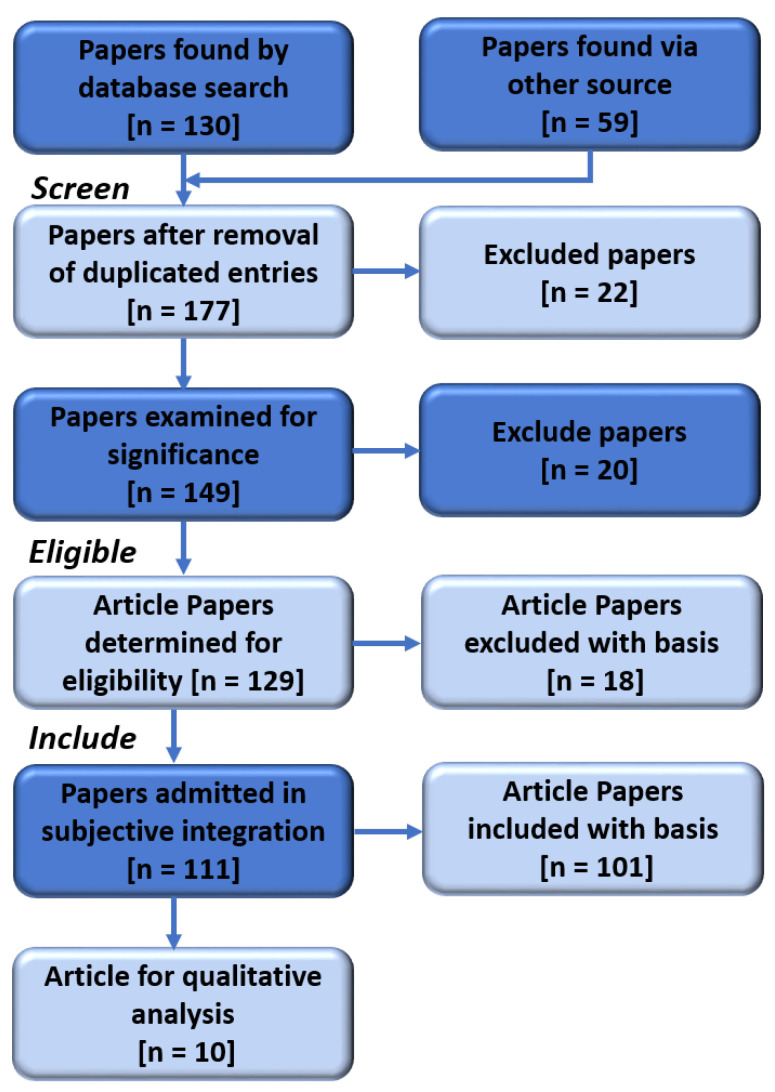
PRISMA flow illustrates how the final 111 papers at the reference were chosen and the 10 publications that specifically addressed combining blockchain with AI for IDS in IoT.

This section presents a methodical illustration utilized for the in-depth evaluation. The ‘mentefacto conceptual design’ [81] and the meta-analysis (PRISMA) [82,83] served as inspiration for the creative reviewing methodologies used in this work. Articles released between 2019 and 2024 were given precedence during the selection process. However, the year of publication becomes inconsequential in the event of a historical context in the evaluation. Ref. [84] states that the following databases are the best places to find research about computer science and engineering: IEEE Xplore, ScienceDirect, Springer, a few social media sites like Academia and Google Scholar, ResearchGate, and Sage. Furthermore, we only considered papers prepared in English for our final evaluation. Table 2 summarizes the lists of documents based on the database source using the critical search terms AI’, ‘CYBERSECURITY’, ‘BLOCKCHAIN’, ‘IDS’, ‘IoT’, and ‘IIoT’. Similarly, Figure 4 displays the flow diagram for PRISMA used for the comprehensive analysis and the criteria for choosing the last set of papers. For IDS, only AI-BLOCKCHAIN integrated documents were used for quantitative analysis. As a result, a thorough narrative analysis enabled a systematic summary and described the findings of the screened literature. The following are the requirements for inclusion of papers in the survey:

1. The articles must be original works released as conference proceedings, journals, or arXiv.
2. The final discussion does not consider background and history; only papers published between 2019 and 2024 are included.
3. Only articles that discuss the problems and challenges of integrating AI-BLOCKCHAIN for cybersecurity are considered for the qualitative study.
4. To be eligible for comparison, this review paper must address blockchain and AI integration for IDS and security compared to other recent review works.
5. English must be used to write all of the papers.
6. Finally, publications with access restrictions are excluded because the writers could not access the databases.

Table 2 and Figure 4 contain the usage summary and document searches, respectively. A total of 189 (130 + 59) publications were found throughout the search. Screening out 22 papers due to duplication left 167. Following a relevancy screening and removing papers with open abstracts but restricted access to the full text, 20 were eliminated. With the above inclusion criteria, 20 of the 129 left documents were eliminated. For the survey, 111 publications in all were used. Of these, 101 were used for qualitative analysis, and the remaining 10 (see Table 3) were only articles on AI and BLOCKCHAIN integration implementation for cybersecurity. Thus, they were used for the particular review.

The rapid IoT expansion, connecting billions of devices, faces several critical challenges, including counterfeit hardware, communication security, system management complexities, and data privacy issues. Although in its infancy, studies have shown that the emergence of blockchain technology, known for its decentralization, transparency, and security, aims to enhance resilience against single points of failure. Immutable records enable transparent data sharing and auditing with cryptographic mechanisms to improve data integrity and authentication while automating processes and enforcing rules within the IoT network. Table 3 highlights some attempts at blockchain-based intrusion detection systems for IoT. The next section spotlights this study’s findings on blockchain applications for IoT intrusion detection.

## 4. Findings and Discussion

### 4.1. Role of Blockchain in Enhancing IDS Security

Blockchain enhances IDS security by ensuring the integrity and reliability of security data through its immutable and decentralized architecture [85,86,87,95]. Timestamping and cryptographically linking data blocks enable secure and tamper-proof logging of IDS events, mitigating the risk of data manipulation or deletion [91,92]. Additionally, blockchain’s distributed consensus mechanism eliminates single points of failure in IDS networks. It provides a transparent audit trail of security events, enhancing reliability and fault tolerance against cyber threats [87,90], and strengthening IDS security. The immutability of blockchain prevents unauthorized alterations once data are recorded, while distributed nodes fortify resilience and resistance against attacks [88,89,94]. Encryption further safeguards identity data, making any attempts to alter data detectable due to the blockchain’s transparent nature [87,93]. Existing implementations and case studies validate blockchain-based decentralized identity management systems, with key players like IBM spearheading advancements in various sectors using blockchain technology [96]. The decentralized identity management systems empower consumers with control over their data, ensuring enhanced privacy, security, and interoperability while fostering a user-centric approach [96]. Overcoming challenges and effectively adopting blockchain can lead to developing safer and more respectful digital identity management systems [96].

Integrating blockchain technology with ML holds immense potential for strengthening security in IoT networks. It offers tamper-resistant and transparent data integrity and transaction verification, which enhances intrusion detection capabilities [16]. This synergy enables real-time monitoring, pattern identification, and anomaly detection, facilitating efficient resource management and proactive maintenance [97]. Combining optimization techniques yields a robust and scalable solution for intrusion detection, boosting efficiency and security in interconnected environments [98]. Improves process efficiency, reducing spatiotemporal scenarios and enabling smart manufacturing processes while facilitating extensive data analysis in industrial IoT networks [12]. Combining blockchain’s immutable nature with ML’s resilience enhances IDS accuracy, trustworthiness, and transparency, ensuring data integrity, eliminating single points of failure, and providing auditable and tamper-proof logs and audit trails for better monitoring and accountability in security incidents.

**Figure 5:**
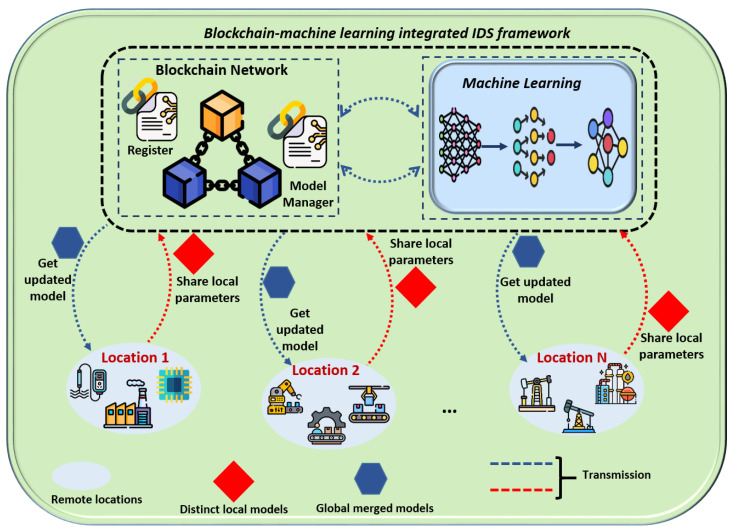
Illustrating the integration of blockchain and machine learning-based intrusion detection.

Some studies have explored the integration of blockchain and ML for intrusion detection, as in Table 3. These attempts showed significance in the various applications. Figure 5 demonstrates the combination of blockchain and IDS in a federated learning approach.

### 4.2. Security of IoT Devices

IoT systems encounter security risks like data breaches and malware due to the sensitive nature of stored information, leading to identity theft and fraud. Exploring IoT and blockchain security delves into blockchain’s potential applications in enhancing IoT security. IoT devices are fundamental components of IoT systems, yet they face significant security challenges due to resource constraints and diverse deployment environments. Authentication and authorization methods for IoT devices in blockchain-based systems must be lightweight and scalable to accommodate limited resources while ensuring security and privacy. Additionally, ensuring firmware integrity and facilitating secure updates are critical but complex tasks in IoT device security, which blockchain smart contracts and consensus mechanisms can address. Moreover, securing communication channels and data privacy and addressing physical security concerns are essential to protecting blockchain-based IoT systems from various threats [2,5,52].

### 4.3. IoT Network Security

In blockchain-based IoT systems, network security is paramount for ensuring system reliability. Challenges include mitigating DDoS attacks, where the distributed network’s nature amplifies risks, and addressing Sybil attacks through identity verification mechanisms. Detecting and mitigating rogue devices is crucial, facilitated by blockchain’s tracking capabilities and anomaly detection algorithms. Additionally, interoperability challenges between IoT devices and blockchain networks necessitate standardized communication protocols and APIs [68]. Furthermore, as IoT device interconnections grow, adopting blockchain can streamline data flow by enabling direct device interactions without centralized servers, enhancing system efficiency and security [5,21,75].

### 4.4. Blockchain Security in IoT

Blockchain technology, fundamental to blockchain-based IoT systems, introduces unique cybersecurity challenges. Consensus mechanisms, crucial for transaction validation, pose challenges due to IoT device constraints, requiring lightweight and energy-efficient solutions. Scalability and performance issues arise from the high transaction volume, demanding scalable blockchain solutions tailored to IoT requirements. Privacy concerns necessitate privacy-preserving techniques like ZKPs, while smart contracts require rigorous security measures to mitigate vulnerabilities. Achieving consensus and governance among diverse stakeholders and navigating regulatory complexities further underscore the multifaceted security challenges in blockchain-based IoT systems [2,51].

IoT devices face security risks like data breaches and malware due to design flaws, interoperability, and remote deployment. Blockchain integration offers encryption, authentication, access control, and vulnerability management to bolster IoT security [2,66]. Although XAI enhances accountability, debugging, adaptation, and deterrence against attacks in blockchain-based IoT systems [83,99,100], additional methodologies such as blockchain, feature attribution, model summarization, counterfactual justifications, and causal modeling enhance system accountability and transparency [100,101]. Despite limitations such as standardization, data privacy, and computational complexity, the potential benefits of XAI in blockchain systems merit further exploration [83,100]. Table 3 summarizes the solutions and challenges across various integration approaches of blockchain for IoT network cybersecurity, highlighting the importance of leveraging blockchain security features to fortify IoT systems against threats and safeguard their operations and data integrity.

### 4.5. Blockchain Application in IoT

The implementation of blockchain has a lot of possibilities to improve the functionality and security of IoT, particularly in smart factories/cities, by ensuring increased trust, transparency, and efficiency [2]. Critical blockchain applications in IoT systems include data integrity and security, achieved through tamper-resistant ledgers distributed across multiple nodes. These are crucial for managing vast data generated in intelligent systems [2,76,80]. Blockchain-based identity management ensures secure access to devices and services by assigning unique cryptographic identities stored on the blockchain, establishing a trustworthy framework for managing IoT ecosystem entities [76,102,103]. Smart contracts automate transactions and processes in smart cities, enabling efficiency and transparency in areas like energy trading and supply chain management [2,55,59]. Blockchain-based IoT networks with decentralized infrastructure and interconnectedness have fewer intermediaries and single points of failure, increasing security and reliability and boosting resilience for industrial applications [55,104,105]. Moreover, blockchain enables micropayments and value exchange between IoT devices, simplifying transactions in scenarios like automated processes in smart factories [77,80]. While this integration promises to revolutionize industrial processes, scalability, interoperability, and governance must be addressed for large-scale IoT deployments [2]. Data transparency and the need for security, availability, and trustworthiness have motivated blockchain technology in IoT. The performance, methodology, and year of publication of a few recent research on blockchain-based IDS for IoT are summarized in Table 3.

The surge in cybersecurity attacks presents a significant challenge for protecting IoT networks due to their inherent vulnerabilities and resource constraints. Integrating IoT with AI has gained traction to bolster security by leveraging AI’s analytical capabilities to detect attack patterns across network traffic [65,78]. However, centralized AI-based approaches face trust and scalability issues, making them incompatible with the decentralized nature of IoT. By facilitating safe data flow between untrusted nodes and offering decentralized defense strategies, blockchain promises to improve IoT security. Despite its potential, blockchain solutions encounter challenges such as limited insight into IoT networks and scalability issues [65,106]. More efficient and intelligent decentralized defense solutions are needed to overcome these hurdles, with AI and blockchain emerging as promising allies, combining AI’s analytical prowess with blockchain’s decentralized architecture.

A blockchain-based training scheme, a secure support vector machine, was presented by Shen et al. [107] to protect IoT data privacy in smart city applications. By leveraging blockchain, the model enables secure data sharing among IoT data providers without depending upon a trusted agent, utilizing a Paillier cryptosystem and encryption methods to guarantee data ownership, integrity, and privacy during training. IoT devices transmit encrypted data to data providers through a unified blockchain ledger, which is stored securely. The proposed approach, evaluated with real-world datasets like the heart disease and breast cancer Wisconsin datasets, maintains SVM classifier accuracy while preserving IoT data privacy.

BlockIoTIntelligence is a framework developed to improve big data analytics by combining blockchain and AI [67]. It consists of four layers: cloud intelligence, fog intelligence, edge intelligence, and device intelligence. Each layer uses blockchain and AI to process and analyze data. The suggested approach proved highly accurate in tackling IoT security, confidentiality, storage capacity, and data flow concerns via qualitative and quantitative analysis, offering a more effective solution than conventional IoT schemes.

### 4.6. Case Study of AI-Blockchain Integration and Result Evaluation

To solve the security and privacy issues with IIoT systems, the works of [89] combine blockchain with AI. In addition, the framework achieves improved classification and detection accuracy with reduced execution time. The anomaly detection performance is improved when auto-encoder-based transformation and blockchain authentication are combined, as seen by the suggested model’s ability to combine high security and computational efficiency compared to alternative methods. However, the major obstacle is the computational complexity of the consensus mechanism.

In [90], researchers utilized blockchain in an FL framework to protect end device data from malicious servers. They introduced a callback mechanism to streamline communication between FL servers and devices, addressing issues like stragglers and dropouts while ensuring secure aggregation of masked models to minimize complexity and resource usage, especially for IoT devices. Despite the computational complexities inherent in blockchain, this method enhanced computational efficiency and ensured secure communication within the federated network. However, using an aggregator/server limits the system to a single point of failure, which is quite concerning.

Blockchain was introduced by Kalapaaking et al. [91] to provide safe FL localized model aggregation in IoT networks. Every blockchain node performed secure aggregation activities using a trusted execution environment (TEE) based on Intel Software Guard Extensions (SGX). Experiments showed comparable processing times to the original FL model and a slight 2% decrease in accuracy. Further practical improvements are needed, such as support for diverse jobs in blockchain-based FL with TEE-based secure aggregation, even though a hash-based consensus mechanism assures model fidelity.

Similarly, a subsequent study by [92] presented a secure and verifiable FL framework for IoT systems, utilizing a TEE and multi-signature scheme to protect the training process and ensure model integrity. Participants trained local models within the TEE, which were verified by the blockchain aggregation manager before aggregation by blockchain nodes. The resulting global model was stored in tamper-proof storage, verified through multi-signature, and distributed to FL participants for subsequent rounds, ensuring the integrity of the FL process. Despite the achievement, the approach still requires enhancing the security and speed of the training process within the secure enclave.

Recently, a study integrated blockchain to secure data privacy during aggregation and transmission, ensuring model update integrity and transparency through smart contracts [46]. This combination minimized global model loss with improved detection accuracy against adversarial perturbation, with average latency for training and aggregation, respectively, enhancing security, scalability, and participant trust.

### 4.7. Practical Implementation and Evaluation Results

Monitoring and optimizing the performance of the IoT systems is essential for intelligent industrial operations, with accurate attack prediction key for effective security management. IoT devices serve as crucial sensors and data collectors in monitoring industrial processes, with real-time data used to train ML models for cybersecurity threat detection, bolstering critical infrastructure security against attacks. However, sharing sensor data centrally raises concerns about privacy and security. FL shows promise in addressing these issues by conducting ML model training on the clients but faces a vulnerability in model parameter aggregation, leading to sensitive information leakage through aggregated model updates.

**Figure 6:**
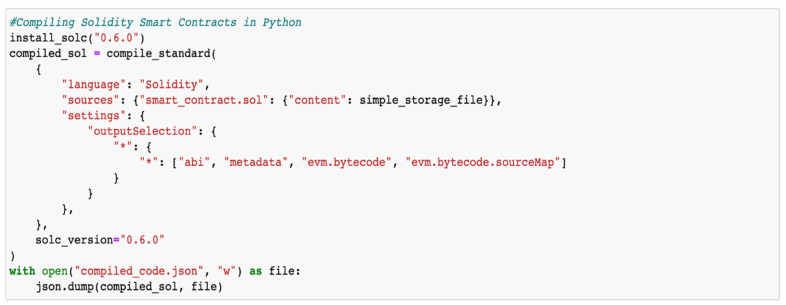
Screenshot showing the compilation of the smart contract.

**Figure 7:**
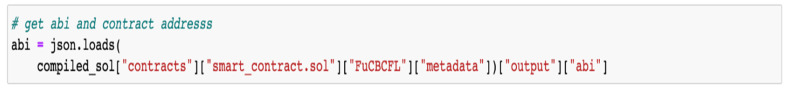
Screenshot showing the connection of the smart contract ABI.

**Figure 8:**
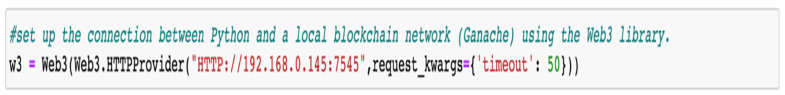
Screenshot showing the connection between Python and Ganache.

**Figure 9:**
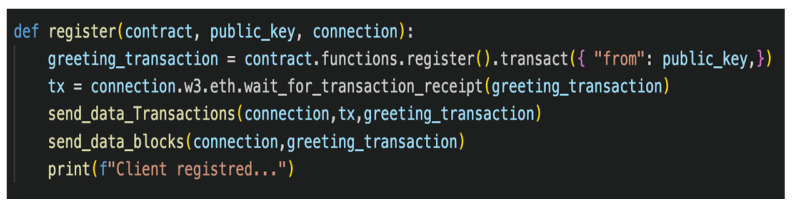
Screenshot showing the blockchain interaction function.

This study includes exploratory results of a blockchain implementation to secure model aggregation to tackle this challenge. The proposed system integrates blockchain with FL, utilizing a private Ethereum platform to establish a blockchain network. FL, executed through a Solidity-based smart contract, incorporates data encryption and aggregation facilitated by the web3.py Python library for blockchain communication. Training involves a convolutional neural network model with enhanced security via adversarial training, fortifying the model against attacks. This methodology combines blockchain platforms, smart contracts, and ML algorithms aided by the application binary interface (ABI), as demonstrated in Figure 6, Figure 7, Figure 8 and Figure 9. This results in a robust FL system with strong privacy guarantees and resistance to adversarial attacks.

The smart contract, built on the Ethereum blockchain platform, automates contract terms enforcement and facilitates the transmission of FL-trained models to the blockchain. Development involved using Remix IDE (https://github.com/ethereum/remix-project, accessed on 1 February 2024) and Solidity linters for accuracy and robustness checks, ensuring adherence to best practices and security standards. Complementing the blockchain, the InterPlanetary File System (IPFS) [108] offers distributed file storage and sharing, enhancing decentralization and data availability. The Web3.py (https://github.com/ethereum/web3.py, accessed on 1 February 2024) Python library enables interaction with the Ethereum blockchain, simplifying smart contract handling and providing tools for development and testing. Challenges in storing model updates on the blockchain due to high gas fees and scalability constraints are addressed by utilizing IPFS for secure storage, minimizing the blockchain’s burden. Interaction functions, such as register(), facilitate client participation in FL by initiating registration transactions, and send_data_transactions() and send_data_blocks() functions ensure transparency by visualizing transaction details and blockchain interaction blocks in the FL process. Table 4 illustrates the performance evaluation of blockchain–FL integration for securing model aggregation against adversarial attacks [46,109].

Simulation results show the possibility of integrating blockchain and FL to enhance secure model aggregation, resulting in higher accuracy and reduced loss values. It supports the advocated strategy of blockchain integration to safeguard model aggregation in FL against adversarial attacks. Efficient data transfer and aggregation are crucial, especially in FL frameworks with blockchain, considering network conditions and model complexity. Network latency becomes a critical factor in scenarios involving dispersed IoT devices.

This study validated its concepts using the Edge-IIoT set (https://ieee-dataport.org/documents/edge-iiotset-new-comprehensive-realistic-cyber-security-dataset-iot-and-iiot-applications, accessed on 2 November 2023) and IoT network intrusion detection (https://ieee-dataport.org/open-access/iot-network-intrusion-dataset, accessed on 2 November 2023) datasets, featuring 62 predictors, 15 classifications, and 157,800 observations in the Edge-IIoT set. The IoT environment covered by these datasets includes a variety of network attack types, including DoS, unauthorized commands, MiTM, reconnaissance, command injection and backdoor attacks, metadata, event data, device identifiers, communication protocols, and regular traffic. Particular sensor data, selected for their applicability in representing communication in a highly vulnerable IIoT network, are included. The sensor data include attributes like IP addresses, ports, protocols, packet length, flow time, and statistics. On an Intel(R) Core(TM) i5-8500 CPU @ 3.00 GHz PC with 8 GB RAM running Windows 11, the experimentation environment consisted of Visual Studio Code, Ganache v2.7.1, Solidity v0.8.22, and Python 3.6.13.

### 4.8. Blockchain-as-a-Service (BaaS): IoT Cybersecurity Perspective

Blockchain-as-a-service (BaaS) in IoT has gained significant attention due to its potential to address cybersecurity concerns [2,50,54,79]. By integrating blockchain technology into IoT networks, BaaS offers several advantages in enhancing cybersecurity:

1. Data integrity and immutability: Providing a tamper-resistant and immutable ledger ensures data integrity stored within IoT networks. Due to the cryptographic links between every transaction on the blockchain and earlier transactions, it is nearly complicated to change past data without the network’s participants’ consent [90].
2. Secure data exchange: Enables safe, direct, peer-to-peer data transfer between IoT devices [46,91,92]. To reduce the danger of data modification or illegal access, smart contracts and programmable self-executing agreements on the blockchain enable automatic and safe data exchanges based on established conditions [46].
3. Decentralization and resilience: Its decentralized architecture eliminates single points of failure, enhancing the resilience of IoT networks against cyberattacks. With no central authority controlling the network, blockchain ensures that data remain accessible even in node failures or malicious attacks [46,91,92,93].
4. Identity and access management: It makes it possible for IoT devices to have strong identification and access control systems, mitigating the risk of unauthorized access and identity spoofing. By confirming the identity of network participants, BaaS improves the security of IoT devices using decentralized authentication procedures [67,107].
5. Audibility and transparency: Real-time auditing of transactions within IoT networks is made possible by the transparent nature of blockchain technology. Data exchange and operations recorded on the blockchain are traceable to their origin, enabling forensic analysis and accountability in case of security breaches [51,89].

While promising solutions are available to improve cybersecurity in the IoT by offering data integrity, decentralized control, and transparency, resolving identified issues is necessary to utilize BaaS in IoT network security fully.

### 4.9. Open Issues and Future Direction

In IoT deployments, devices’ limited computational power, memory, and energy pose challenges for integrating blockchain solutions [110]. Running full blockchain nodes on resource-constrained devices is impractical, and BaaS introduces additional latency and strain on such devices due to network communication and consensus requirements [110]. Moreover, the transparency of public blockchains raises privacy concerns, necessitating privacy-preserving techniques for sensitive IoT data [79]. Integrating BaaS with IoT systems requires careful planning to address interoperability and data synchronization complexities [79]. As researchers explore lightweight cryptographic schemes and efficient integration methods, it is crucial to balance the security benefits of blockchain with the computational demands imposed on IoT devices.

While BaaS offers benefits for IoT, it faces challenges in scalability, consensus mechanisms, and latency, given the volume of interconnected devices. The overhead costs of blockchain operations, including transaction fees and infrastructure, may limit its feasibility in resource-constrained IoT environments. Additionally, the immutable nature of blockchain transactions raises privacy concerns, necessitating privacy-preserving techniques like ZKPs or private blockchains to protect sensitive IoT data [2,54,79].

Layer-2 protocols, such as lightning networks, boost blockchain scalability by handling transactions off-chain and settling periodically on the main chain. Bridging diverse blockchains via standards like Polkadot and Cosmos fosters seamless communication among IoT networks [54,65]. PoS offers energy efficiency compared to PoW, which is particularly beneficial for power-intensive IoT mining activities [54,111]. Directed acyclic graphs provide scalability and energy efficiency, aligning well with IoT needs. Hybrid models merging blockchain with traditional databases ensure security and efficiency by storing metadata on-chain while keeping raw data off-chain [54]. Blockchain-driven edge computing enables edge devices to manage a lightweight blockchain locally, reducing latency and enhancing privacy.

The ongoing advancement of quantum computing underscores the importance of fortifying blockchain against quantum attacks through research on quantum-safe cryptographic algorithms [69,70,71]. Addressing blockchain’s energy consumption requires focusing on energy-efficient consensus protocols like PoS or PoA [54]. Scalability solutions such as sharding, sidechains, or off-chain protocols are critical for large-scale IoT deployments. Future exploration areas include interoperability standards, privacy-preserving mechanisms like ZKPs, and integrating AI/ML with blockchain for robust cybersecurity [54,65]. Real-world testing, regulatory compliance frameworks, and collaborative industry efforts are pivotal for validating and implementing blockchain-based IoT security solutions [2,54].

This study reveals that to uphold blockchain integrity and security against quantum threats, it is crucial for blockchain platforms to seamlessly integrate algorithms like PQC while upgrading cryptographic primitives like digital signatures, key exchange protocols, and hash functions to PQC standards [71,72,73]. There is a need to maintain reverse collaboration to enhance transaction efficiency. Collaboration among blockchain developers, cryptographers, and quantum computing specialists is necessary to navigate this quantum transition safely [71,72,73].

## 5. Conclusions

This paper comprehensively reviews blockchain applications for cybersecurity in IoT networks, presenting a robust framework for integrating blockchain into IDS within IoT networks and addressing crucial research avenues, current trends, and notable challenges. The study illuminates emerging areas in IoT security through a systematic analysis of articles spanning AI, blockchain, IDS, IoT, and IIoT. By evaluating recent advancements and diverse AI blockchain integration methods, this research underscores the pivotal role of blockchain in bolstering intrusion detection performance. This framework offers a roadmap for collaborative exploration, aiming to advance IDS for universally accessible, scalable, transparent, immutable, and decentralized IoT networks, driving innovation in IoT cybersecurity. Simulation results from highlighted case studies demonstrate that—despite resource constraints and privacy issues—blockchain’s intense presence in IoT networks ensures ongoing progress toward a more secure and resilient IoT landscape, necessitating further research into lightweight cryptography, efficient consensus mechanisms, and privacy-preserving techniques to overcome existing barriers.

## Figures and Tables

**Table 1:** Summary of the review of the literature highlighting the key findings of each paper (Yes: *√*, No: χ).

Author	Year	Systematic Review Methodology	IoT	Blockchain Integration	IDS	Use Case Demonstration
[75]	2018	χ	*√*	χ	χ	χ
[53]	2019	χ	*√*	χ	χ	χ
[74]	2019	χ	*√*	*√*	χ	χ
[68]	2019	*√*	*√*	χ	χ	χ
[54]	2019	χ	*√*	χ	χ	χ
[76]	2020	χ	χ	χ	χ	χ
[77]	2020	χ	*√*	*√*	*√*	χ
[78]	2020	χ	*√*	*√*	χ	χ
[55]	2021	χ	*√*	*√*	χ	χ
[58]	2021	χ	*√*	χ	χ	χ
[12]	2022	*√*	*√*	*√*	*√*	χ
[38]	2022	χ	*√*	χ	*√*	χ
[57]	2022	χ	*√*	χ	χ	χ
[56]	2022	χ	*√*	χ	χ	χ
[52]	2022	χ	*√*	*√*	χ	χ
[65]	2022	*√*	*√*	*√*	χ	χ
[79]	2022	χ	*√*	*√*	χ	χ
[21]	2023	χ	*√*	*√*	χ	χ
[80]	2023	χ	*√*	*√*	*√*	χ
[59]	2024	*√*	*√*	*√*	*√*	χ
*This Study*	2024	*√*	*√*	*√*	*√*	*√*

**Table 2:** Publications employed in this study.

Database Source	No. of Documents	% Freq
IEEE Xplore (Journals)	30	27.03
IEEE Xplore (Conferences)	10	9.01
MDPI	18	16.22
Springer	18	16.22
ACM	3	2.70
arXiv Pre-print	2	1.80
Google Scholar	5	4.50
Hindawi	1	0.9
Frontiers	2	1.80
Taylor & Francis	1	1.09
ScienceDirect (Elsevier)	12	10.81
Other Sources (Blogs, Reports, and Websites)	10	9.01
Total	111	100.00

**Table 3:** Articles on blockchain-based IDS techniques for IoT/IIoT.

Study	Technique	Focus	Achievement	Year
[85]	Proposed a combination of blockchain and CNN for Software-defined network (SDN)-based IIoT architectures	To detect and prevent security threats in the application and network security layers of the SDN-based IIoT architectures.	Minimized the impact of attacks on SDN-based IIoT architecture layers.	2023
[86]	Creation of an IDS powered by ML algorithms and blockchain to improve the privacy and security of IoT devices.	Aims to encrypt interactions between IoT devices.	Simulation results could improve privacy and security by providing a tamper-proof decentralized communication system.	2023
[87]	Deep learning with blockchain orchestration for safe data transfer in IoT-enabled healthcare systems.	The approach ensures secure data transmission and integrity by exploiting the zero-knowledge proof (ZKP) scheme	Using the Ethereum smart contract to handle data security concerns with the interplanetary file system (IPFS) for off-chain storage to alleviate the problem of data storage costs.	2023
[88]	A hybrid decision tree method	To integrate ML with blockchain for anomaly detection	Predict attack within the shortest time with high detection accuracy.	2023
[89]	A lightweight blockchain security model driven by AI.	To guarantee the security and privacy of cloud-based IIoT systems.	Improved performance in anomaly detection when compared with other models.	2023
[90]	A secure aggregation mechanism for FL based on blockchain	By ensuring secure aggregation, local device data masking stops hostile servers from compromising and reconstructing training data.	The technique minimizes resource waste and quickens the global model’s convergence rate by synchronizing clients with an antiquated model.	2023
[91]	A blockchain network is used in the proposed system for a safe FL model aggregation.	To safely carry out the FL-based aggregation and produce a global model.	According to experimental results, the framework’s processing time was nearly identical to that of the original FL model.	2023
[92]	Multi-signature authentication is used to confirm the integrity of the global ML model and TEE is used to safeguard each client’s local model training.	To give a verifiable ML model and guarantee the participant’s local model training security.	The training on the secure enclave resulted in a slight drop in accuracy, according to the experimental findings. Additionally, multi-signature execution time has no discernible impact on blockchain network speed.	2023
[93]	A blockchain-driven edge intelligence methodology	Incorporates blockchain based on a reputation for decentralized transaction recording and verification, guaranteeing privacy and data protection.	The simulation findings validate the approach’s efficiency and robustness over state-of-the-art cyberattack detection methods.	2022
[94]	A security architecture that combines SDN and blockchain technology.	To defend industrial control processes from counterfeit commands and stop misrouting attacks on OpenFlow rules in industrial IoT systems with SDN enabled.	The assessment’s findings confirm the suggested security measures’ effectiveness and efficiency.	2019

**Table 4:** Comparison of the blockchain–FL integration performance between Edge-IIoT and IoT Network intrusion datasets.

	Edge-IIoT Dataset						IoT Network Intrusion Dataset					
	**Local Model**			**Global Model**			**Local Model**			**Global Model**		
**Clients**	**Accuracy** **(%)**	**Loss**	**Train** **Time (s)**	**Accuracy** **(%)**	**Loss**	**Aggregation** **Time (s)**	**Accuracy** **(%)**	**Loss**	**Train** **Time (s)**	**Accuracy** **(%)**	**Loss**	**Aggregation** **Time (s)**
1	81.72	0.7359	580	84.80	0.5895	501	94.11	0.2205	486	95.74	0.1551	401
2	82.59	0.7538	572	85.09	0.5892	555	93.88	0.1685	475	95.68	0.1579	445
3	80.69	0.7475	563	84.77	0.5873	564	93.82	0.2270	464	95.47	0.1638	456
4	79.60	0.7619	420	84.97	0.5871	599	94.03	0.2215	388	95.57	0.1603	548
5	82.79	0.7326	389	84.80	0.5990	530	93.84	0.2311	400	95.94	0.1568	509

## Data Availability

No new data were created or analyzed in this study. Data sharing is not applicable to this article.

## Abbreviations

The following abbreviations are used in this manuscript:

AI	artificial intelligence
ABI	application binary interface
API	application programming interface
ANN	artificial neural network
BaaS	blockchain-as-a-service
CNN	convolutional neural network
CRNN	convolutional and recurrent neural network
DDoS	distributed denial-of-service
DL	deep learning
DoS	denial-of-service
DPoS	delegated proof of stake
ECC	elliptic curve cryptography
FL	federated learning
HMI	human–machine interface
ICS	industrial control system
IDE	integrated development environment
IED	intelligent end device
IDS	intrusion detection system
IoT	Internet of Things
IIoT	Industrial Internet of Things
IPFS	interplanetary file system
LOIC	Low Orbit Ion Cannon
MiTM	man-in-the-middle
ML	machine learning
MTU	master terminal unit
MSU	master station unit
OTA	over-the-air
OSI	open systems interconnection
PBFT	practical Byzantine fault tolerance
PLC	programmable logic controller
PoA	proof of authority
PoS	proof of stake
PoW	proof of work
PQC	post-quantum cryptographic
PRISMA	preferred reporting items for systematic reviews and meta-analyses
RFID	radio frequency identification
RNN	recurrent neural network
RSA	Rivest–Shamir–Adleman
RSU	remote station unit
RTU	remote terminal unit
SCADA	supervisory control and data acquisition
SDN	software-defined network
SGX	Software Guard Extensions
SVM	support vector machine
TEE	trusted execution environment
XAI	explainable AI
ZKP	zero-knowledge proof
